# Exercise Experiences and Changes in Affective Attitude: Direct and Indirect Effects of *In Situ* Measurements of Experiences

**DOI:** 10.3389/fpsyg.2016.00900

**Published:** 2016-06-16

**Authors:** Gorden Sudeck, Julia Schmid, Achim Conzelmann

**Affiliations:** ^1^Institute of Sport Science, University of TübingenTübingen, Germany; ^2^Institute of Sport Science, University of BernBern, Switzerland

**Keywords:** physical exercise, affective response, perceptions of competence, perceived exertion, affective attitudes toward exercise

## Abstract

**Objectives:** The purpose of this study was to examine the relationship between exercise experiences (perceptions of competence, perceived exertion, acute affective responses to exercise) and affective attitudes toward exercise. This relationship was analyzed in a non-laboratory setting during a 13-weeks exercise program.

**Materials and Methods:** 56 women and 49 men (aged 35–65 years; *M*_age_ = 50.0 years; *SD* = 8.2 years) took part in the longitudinal study. Affective responses to exercise (affective valence, positive activation, calmness) as well as perceptions of competence and perceived exertion were measured at the beginning, during, and end of three exercise sessions within the 13-weeks exercise program. Affective attitude toward exercise were measured before and at the end of the exercise program. A two-level path analysis was conducted. The direct and indirect effects of exercise experiences on changes in affective attitude were analyzed on the between-person level: firstly, it was tested whether perceptions of competence and perceived exertion directly relate to changes in affective attitude. Secondly, it was assessed whether perceptions of competence and perceived exertion indirectly relate to changes in affective attitudes—imparted via the affective response during exercise.

**Results and Conclusion:** At the between-person level, a direct effect on changes in affective attitude was found for perceptions of competence (β = 0.24, *p* < 0.05). The model revealed one significant indirect pathway between perceived exertion and changes in affective attitude via positive activation: on average, the less strenuous people perceive physical exercise to be, the more awake they will feel during exercise (β = -0.57, *p* < 0.05). Those people with higher average levels of positive activation during exercise exhibit more improvements in affective attitudes toward exercise from the beginning to the end of the 13-weeks exercise program (β = 0.24, *p* < 0.05).

Main study results have revealed that *in situ* experiences predicted changes in affective attitude during multi-week exercise programs. These relevant *in situ* experiences encompass cognitive factors, the sensation of interoceptive cues, and affective responses to exercise. Considering the predictive role of affective attitudes for exercise behavior, these findings suggest that exercise interventions should put greater emphasis on specific exercise experiences.

## Introduction

The positive effect of physical exercise on health has already been demonstrated (Physical Activity Guidelines Advisory Committee Report, 2008). Nevertheless, surveys among the general population show that the majority of adults in Western industrialized countries do not exercise enough (European Commission, 2014). Encouraging participation in exercise is therefore an important assignment for society and policy-makers.

In order to create effective interventions, it is crucial to identify factors and mechanisms associated with the initiation and maintenance of physical exercise behavior (e.g., Michie and Abraham, 2004; Biddle and Fuchs, 2009). Several psychological theories have been developed that feature prominently in explaining health behavior [e.g., social cognitive theory (Bandura, 1997); theory of planned behavior (Ajzen, 1988); health action process approach (Schwarzer, 2008)]. In recent years, these theories have been advanced in the domain of physical exercise.

Particularly, research has placed a stronger focus on the role of affective processes in initiating and maintaining regular physical exercise. For example, existing studies based on the theory of planned behavior have for the most part predicted exercise behavior via instrumental attitudes (e.g., cognitive convictions about the benefits of physical exercise, Lawton et al., 2009). More recent work, however, has shown that affective attitudes toward exercise (i.e., whether thinking about doing physical exercise makes one feel comfortable or uncomfortable) is at least as successful at explaining exercise behavior (e.g., Lowe et al., 2002; Brand, 2006; Kiviniemi et al., 2007). Further evidence for the particular relevance of affective associations to exercise could be drawn from recent studies that differentiate between distal and proximal outcome expectancies. Outcome expectancies are closely related to affective attitudes toward exercise when they are proximal to the behavior itself and refer to affective states (e.g., “When I exercise, then I will feel better”). These proximal outcome expectancies predict exercise intention and behavior to a greater degree than distal outcome expectancies like future health benefits (Gellert et al., 2012). Furthermore, the fulfillment of proximal outcome expectancies (e.g., in terms of emotional reward) provides a crucial mechanism for long-term exercise change as it supports volitional effort in goal-directed behavior (Klusmann et al., 2016).

Moreover, recent research indicates that exercise experiences need to be better integrated with advancements in exercise behavior theories. Psychological and physical experiences appear to be especially relevant for the longer-term maintenance of exercise behavior (Rothman, 2000). There is some initial empirical support that positive exercise experiences influence subsequent affective outcome expectancies (Loehr et al., 2014) or satisfaction with the exercise behavior (Fleig et al., 2011; Baldwin et al., 2013).

One of the few models that integrate both exercise experiences and (affective) attitudes is the transdisciplinary framework model put forward by Bryan et al. (2011). They assume that exercise experiences, such as perceived exertion or changes in affect, influence attitudes, which in turn predict exercise behavior. However, the model is heuristic in nature. Experiences, for instance, are aggregated into a single category, and possible relationships between different experiences are not put into concrete terms. Furthermore, it is unclear *which* exercise experiences alter affective attitudes. This represents the starting point for this paper, which aims to investigate the mechanisms operating between exercise experiences and changes in affective attitude.

### Changes in Affective Attitude: Potential Mechanisms

**FIGURE 1:**
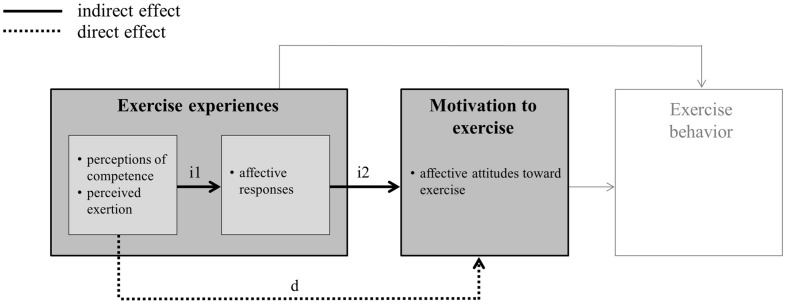
**Conceptual model of changes in affective attitude toward exercise (based on Bryan et al., 2011)**.

Existing intervention studies on changes in affective attitude toward physical exercise support the assumption that affective attitudes are influenced by one’s personal experiences with exercise (Rhodes et al., 2009). Thus, Williams (2008) assumed that specific exercise experiences influence affective responses to physical exercise, and these in turn alter affective attitudes and favor longer-term exercise adherence (see **Figure 1**, indirect effect). On the one hand, Williams (2008) draws on dual mode theory (e.g., Ekkekakis and Acevedo, 2006), which explains different affective responses to exercise activities, firstly in terms of cognitive aspects. One cognitive factor often examined in this context are perceptions of competence (e.g., Rose and Parfitt, 2007). Positive perceptions of competence arise when a (motoric) task can be performed using one’s own skills and abilities. A positive connection between this and affective responses has been repeatedly demonstrated (e.g., Rose and Parfitt, 2012). Secondly, dual mode theory explains different affective responses to exercise activities in terms of interoceptive cues. Interoceptive cues, which may be triggered by physical exercise, include such sensations as having difficulty breathing or increased body temperature. A global indicator for experiencing interoceptive cues is perceived exertion. This again is defined as “a configuration of sensations: strain, aches and fatigue involving the muscles and the cardiovascular and pulmonary systems during exercise” (Groslambert and Mahon, 2006, p. 912). There is empirical evidence which confirms that perceived exertion is negatively connected to affective responses to physical exercise (e.g., Williams et al., 2008; Barnett, 2013). On the other hand, Williams also drew on hedonistic theories, which state that human beings are programed by evolution to seek pleasure and avoid pain (Kahnemann, 1999). Experiencing pleasure while exercising forges the corresponding affective associations, which in turn reinforce the repetition of exercise behavior.

Aside from an indirect link between exercise experiences and changes in affective attitude, a direct connection is also feasible. For example, Vlachopoulos and Michailidou (2006) were able to show that perceived competence is linked to affective attitudes (see **Figure 1**, direct effect). This indicates a direct relationship between the cognitive appraisal of one’s own abilities and affective attitudes toward exercise. However, we did not find studies on the connection between perceived exertion and changes in affective attitude during exercise activities.

### Affective Responses to Exercise in the Behavior Change Research

Affective responses to exercise are connected to motivation and exercise behavior (e.g., Kwan and Bryan, 2010; Williams et al., 2012). When studying these relationships, three conceptual and methodological issues need to be considered:

(1) The dimensional approach of basic affect: the conceptualization and measurement of affective responses to exercise are either based on a categorical or a dimensional approach. According to a categorical approach affective states are organized into distinct categories. Researchers often use several distinct categories, such as depression, tension, confusion, anger, fatigue, vigor, or anxiety. According to a dimensional approach affective states are systematically interrelated. Therefore, they can be described using only a few basic affect dimensions. There is some empirical support for the three dimensions affective valence (e.g., discontent – content), positive activation (e.g., tired – awake) and calmness (e.g., tense – relaxed; Schimmack and Grob, 2000). In the present study affective response is examined from a dimensional approach. Since it is unclear, which specific affective state is the most important for physical exercise motivation, a broad perspective is more likely “to capture the essence of exercise-induced affect” (Gauvin and Brawley, 1993, p. 152). An additional advantage of the dimensional approach is that affective states can be measured more efficiently. The measurement of a few basic dimensions using only a few reliable items per dimension is particularly suitable for study designs with frequently repeated measurements (Ekkekakis, 2008).
(2) Time of affect measurement: affective responses vary depending on the time at which affect is measured (Ekkekakis et al., 2011). Affective responses during physical exercise display a greater inter-individual variability than those occurring afterward. If affective states are only determined before and after exercise, there is the danger that one will actually measure the response to finishing the activity, which is usually positive (Hall et al., 2002; Backhouse et al., 2007). Possibly, it is this high inter-individual variability during exercise which is particularly significant for explaining the various motivations for exercising (Kwan and Bryan, 2010).
(3) Intra-individual differences in affective responses to exercise: in the behavior change research, the majority of studies measure affective responses to exercising during a single exercise session (Kwan and Bryan, 2010; Williams et al., 2012). Thus, intra-individual consistency in affective responses is rarely taken into account. A recent study with overweight and obese women demonstrated that pre-to-post-exercise changes in affect within an individual varied across three identical, moderate-intensity treadmill exercise sessions (ICCs = 0.02–0.60, Unick et al., 2015). In contrast, affective responses during exercise were more consistent (ICC = 0.72), especially when controlling for pre-exercise scores (ICC = 0.83).

In sum, current research indicates that it is important to assess affective responses especially *during* exercise. To obtain reliable information about affective responses to exercise, they should be measured more than once. Additionally, existing studies often examined physical exercises in controlled laboratory conditions. Thus, whether affective responses to exercise in a laboratory setting are representative of a participant’s real-world affective response remain unclear (Liao et al., 2015).

### Aims and Rationale of the Present Study

The aim of the present study is to examine the relationship between exercise experiences and changes in affective attitude (see **Figure 1**). In the category of experiences, perceptions of competence and perceived exertion are examined, as well as affective responses to physical exercise. Following the example of Williams (2008), we assume that the perceptions of competence (positively) and perceived exertion (negatively) impact affective attitudes as partially mediated via affective responses to exercise. This indirect path of operation is compared with a direct path of operation, in which it is evaluated whether perceptions of competence and perceived exertion directly predict changes in affective attitude.

Compared to the current state of the research, the present study stands out due to the following extensions:

(1) Exercise experiences are measured *in situ* during exercising. In previous studies, exercise experiences were measured retrospectively—for example, concerning the period of the last few weeks (Fleig et al., 2011; Parschau et al., 2013). Moreover, in some diary studies exercise experiences were recorded on a weekly basis (Loehr et al., 2014) or on a daily basis (Baldwin et al., 2013). The latter studies revealed that it is especially worthwhile to assess exercise experiences as close as possible to the actual activities in order to gain insights into their specific qualities.
(2) Exercise experiences are measured in a non-laboratory setting. In doing so, variables can be measured closer to usual, naturalistic conditions than it is done in other current studies with controlled laboratory conditions.
(3) The three basic affective dimensions—affective valence, positive activation, and calmness—are simultaneously integrated into our analysis. Thereby, our study helps clarify which affective dimensions have an effect on affective attitudes toward exercise.
(4) As affective responses to exercise within an individual vary across different exercise sessions (Unick et al., 2015), observations of exercise experiences in multiple exercise sessions provide a reliable assessment of exercise experiences in the present study. In order to consider personal characteristics of the participants, we included physical fitness as control variable for affective responses during exercising (Ekkekakis et al., 2011).
(5) Affective attitudes are measured twice, 13 weeks apart, and are modeled as a latent change (LC) variable in the analyses (Reuter et al., 2010). This allows us to adequately model the dynamics of this connection: exercise experiences should be associated with *medium-term change* in affective attitude.

As previously stated, we test the assumptions made by Williams (2008) and, therefore, compare indirect and direct associations between inter-individual differences in exercise experiences and changes in affective attitude. However, repeated measurements of exercise experiences make it possible to consider both inter-individual (between-person) variations and intra-individual (within-person) variations in the features under study. Therefore, we chose a multi-level approach and applied a two-level path analysis. The multi-level approach is of particular relevance for the first part of the indirect effects under study (see **Figure 1**). The links between exercise experiences and affective responses to exercise can have different meanings depending on whether between-person variations or within-person variations are being analyzed (e.g., Hamaker, 2012). Considering, for example, a negative relationship between perceived exertion and affective responses to exercise on the between-person level would mean that persons who experience on average a higher level of perceived exertion when exercising would have less positive affective responses. In contrast, the same negative relationship on the within-person level would mean that a given individual in different exercise sessions would exhibit a more negative affective response if the activity were perceived as being more strenuous. If the decomposition of between-person effects and within-person effects were ignored and only between-person effects were considered, an improper generalization of between-person effects could occur. The between-person relationship would be inappropriately generalized to intra-individual relationships between perceived exertion and affective valence. Against this background, it is important to note that the primary focus of the present study is on inter-individual differences in exercise experiences and changes in affective attitude (between-person effects). However, considering both within-person effects and between-person effects minimize the risk of possible misinterpretations of the results for the first part of the indirect pathway on the between-person level, which is of primary interest for the confirmatory hypotheses of the present study.

Aside from this confirmatory test of the hypotheses, this study also includes explorative parts. For instance, no *a priori* assumptions were made as to *which* of the three affective dimensions is connected to perceptions of competence and perceived exertion. Moreover, no assumptions were made about within-person associations between exercise experiences and affective responses to exercise due to the lack of previous studies that have examined within-person variations of exercise experiences during exercise. Finally, no assumptions were made about *which* of the three affective dimensions, has a direct effect on changes in affective attitude.

## Materials and Methods

The present study is based on data from the research project “Which sport for whom?” This project was a quasi-experimental intervention study investigating the effectiveness of five newly created exercise programs as regards promoting subjective well-being and exercise behavior.

### Participants

The sample consisted of academic and non-academic staff from a university in the German-speaking part of Switzerland. It comprised of 80 women and 53 men. Their average age was 49.5 years (*SD* = 8.3). Most were married (61%), and their highest level of education was a degree from a university or a university of applied science (65%). A survey based on a 7-days recall procedure (Mäder et al., 2006) revealed that, at the beginning of the study, 23% engaged in no exercise at all and 6% exercised for less than 1 h per week; 21% exercised for 1–2 h per week; 23% exercised for 2–4 h per week and 26% for more than 4 h. The objectively measured body mass index showed that 67% had normal body weight, whereas, 24% were overweight and 9% obese (see **Table 1**).

**Table 1:** Sample characteristics.

		Total sample (*n* = 133)		Reduced sample (*n* = 105)	
		*n*	%	*n*	%
Sex	Male	53	39.8	49	46.7
	Female	80	60.2	56	53.3
Marital status	Married	73	61.3	59	62.8
	Unmarried	27	22.7	20	21.2
	Divorced/separate	19	16.0	15	16.0
	Not available	14	-	11	-
Highest education	Professional honor	19	16.2	13	14.3
	College of professional Education and training	18	14.5	13	13.3
	University or university of applied science	80	64.5	66	67.3
	Other	6	4.8	5	5.1
	Not available	9	-	7	-
Exercise behavior	No exercise	30	23.4	24	23.8
	<1 h/week	8	6.3	7	6.9
	1–2 h/week	27	21.1	21	20.8
	2–4 h/week	30	23.4	23	22.8
	>4 h/week	33	25.8	26	25.7
	Not available	5	-	4	-
Body weight	Normal	73	67.0	57	67.1
	Overweight	26	23.9	22	25.9
	Obese	10	9.1	6	7.0
	Not available	24	-	20	-
Exercise programs	Active and recreated	20	15.2	16	15.4
	Reload and relax	26	19.7	18	17.3
	Together fit	13	9.8	13	12.5
	SPORT Varia	32	24.2	26	25.0
	Body and (e)motion	42	31.1	32	29.8
Age		*M* = 49.5		*M* = 50.0	
		*SD* = 8.3		*SD* = 8.2	

### Instruments

#### Affective Attitudes toward Exercise

To measure affective attitudes toward exercise, we applied a questionnaire by Crites et al. (1994), whose German-language version was validated by Brand (2006). This instrument comprises four items based on the phrase: “When I think about exercising, I feel…” Answers follow a semantic differential of values from 1 to 9. The answers to the four items are: *“not relaxed”* – *“extremely relaxed”* (AFF1); *“not satisfied”* – *“extremely satisfied”* (AFF2); *“not happy”* – *“extremely happy”* (AFF3); *“not uncomfortable”* – *“extremely uncomfortable”* (AFF4). This last item proved to have a comparatively low discriminatory power due to the double negative (pre-test: *r_it_* = 0.50; post-test: *r_it_* = 0.50) and was therefore removed from the scale. The internal consistency of the remaining three items was good in the existing sample (pre-test: α = 0.82; post-test: α = 0.92).

#### Affective Response to Exercise

Affective states were measured using Wilhelm and Schoebi’s (2007) short, German-language scale for assessing the three basic dimensions affective valence, positive activation, and calmness (Schimmack and Grob, 2000). Validating studies have shown that these three bipolar dimensions can be reliably established by means of the corresponding two items (Wilhelm and Schoebi, 2007). Bipolar pairs of adjectives served as indicators to register affective valence (*“discontented”* – *“contented,” “unwell”* – *“well”*), calmness (*“tense”* – *“relaxed,” “agitated”* – *“calm”*) and positive activation (*“tired”* – *“awake,” “full of energy”* – *“without energy”*). For each pair of adjectives, participants had to answer the question “At this moment, I am feeling...” on a seven-point scale ranging from 0 to 6. In our own sample, the inter-item correlations within the three dimensions were satisfactory to good (affective valence: 0.58 ≤*r* ≤ 0.74; calmness: 0.61 ≤*r* ≤ 0.68; positive activation: 0.59 ≤*r* ≤ 0.76).

#### Perceived Exertion

Ratings of perceived exertion were established with reference to a validated, German-language scale by Buskies and Boeckh-Behrens (2000). This instrument is a simplified version of Borg’s (1982) scale for rating perceived exertion. The participants answered the question: “How exerting did you find the activity?” on a seven-point scale, with answers expressed in words [(1) *“very light,”* (2) *“light,”* (3) *“rather light,”* (4) *“moderate,”* (5) *“somewhat hard,”* (6) *“hard,”* (7) *“very hard”*]. Buskies and Boeckh-Behrens recommend this procedure with less experienced exercisers when preparatory exercises for answering a differentiated Borg-scale are not possible.

#### Perceptions of Competence

We introduced a single item to establish perceptions of competence. This item was phrased with reference to the sub dimension “challenge-skill balance” from the flow state scale (Jackson and Marsh, 1996). The question: “When you think about today’s exercise session: what experience have you gained?” was to be answered using the statement: “The feeling of being competent enough to fulfill the demands of the exercise session” on a six-point rating scale (0 *“not at all true”* up to 5 *“completely true”*).

#### Perceived Physical Fitness

The level of physical fitness was measured by subjective ratings. We applied a modified version of the subscale “physical fitness” from the adjective list for assessing the perceived physical state (PEPS; Kleinert, 2006; Schneider et al., 2010). Participants were asked to spontaneously judge to what extent they feel “strong,” “fit,” and “enduring.” They had to answer on a six-point rating scale (0 *“not at all”* up to 5 *“totally*”). The internal consistency for this modified subscale was good (0.82 *≤* α ≤ 0.88; see also Sudeck and Conzelmann, 2014). The mean score of the three items was used in the further analyses.

### Procedures

In order to recruit the participants, letters were sent to all university employees by post or by e-mail. They were informed that the university sports department was going to offer its employees between the ages of 35–65 years five different new exercise programs.

In brief, the titles and contents of the exercise programs were:

(a) Active and recreated: a combination of endurance and strength training without equipment; short sequence of active relaxation exercises at the end of a session (5–10 min).
(b) Reload and relax: various fitness activities; separate relaxation period at the end of each session (15–20 min).
(c) Together fit: health-oriented aerobic endurance and strength training combined with little games; sociable, playful setting.
(d) SPORT Varia: a combination of sports games and endurance activities; sociable formats.
(e) Body and (e)motion: dancing and rhythmic activities, music-oriented fitness training.

If an employee was interested, they filled in a written application to attend one of the exercise programs and agreed to participate in the study, after being informed about its goals and procedure in line with the Declaration of Helsinki. The ethics commission of the Faculty of Humanities of the University of Bern evaluated the study design and procedures as ethically unproblematic.

**FIGURE 2:**
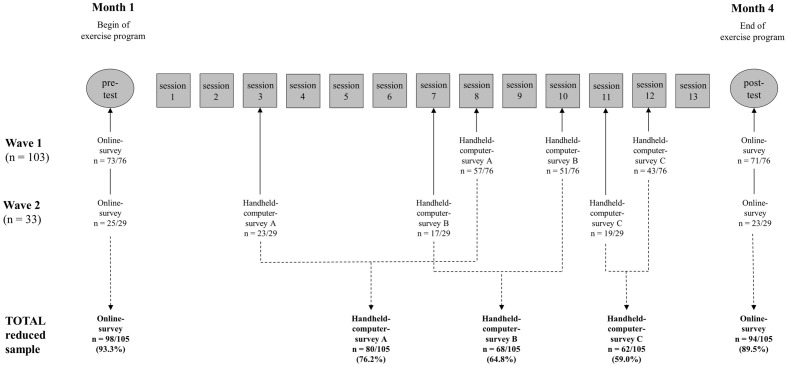
**Study design, procedures, and flow of participants**. Not considered in the reduced sample: *>n* = 23 (no data for at least one handheld-computer-survey; missed all three possible sessions); *n* = 5 (no data for pre-test or post-test).

Affective attitudes toward exercise was assessed at the beginning (pre-test at month 1; see **Figure 2**) and at the end of the intervention period (post-test in month 4). To do this, we set up online questionnaires and sent the participants links by e-mail. A personal registration number enabled us to collate an individual’s data pseudo-anonymously.

Program sessions were held once a week during a 13-weeks intervention period. Each session lasted 60 min. The first 40–50 min of the sessions were relatively similar in all programs. Physical exercises with a predominantly moderate to strenuous intensity were carried out and the exercises were guided and carried out in groups. For this reason, it is only the affective responses during these first 40–50 min that are of interest for the primary research question.^1^ Affective states, perceived exertion, perceptions of competence, and the perceived fitness level were assessed in three out of the 13 exercise sessions. For these three sessions, all trainers were asked to realize typical sessions. Affective states were measured immediately before the exercise session and twice during the session (approximately between minutes 10 and 15, as well as between minutes 40 and 50). The perceived fitness level was assessed before the session. The perceived exertion was assessed twice during each exercise session. Since the regular course sessions ought to be only minimally interrupted out of respect for the course instructors and participants, we dispensed with collecting further survey content during the session. For this reason, perceptions of competence was measured only once at the end of the exercise session. These assessments were carried out using the software IzyBuilder 2.0 on a handheld computer (HP iPAQ114). In all three sessions, a trained student was present. He gave a hand signal to the participants when it was time to fill out the surveys and provided assistance in case of any problems with the handheld computers.

The study was carried out in two waves. Of the 133 subjects in total, 103 took part in one of the five exercise programs for the first time in wave one, and 30 in wave two. In order not to burden participants with immediate data collection, the handheld computer surveys took place at intervals of 2–4 weeks. For organizational reasons, it was not possible to carry out the three handheld computer surveys in wave one and two in the same course weeks (see **Figure 2**).

The following analyses are based on a reduced sample of 105 individuals (see **Table 1**). Twenty-three people were excluded from the calculations because no handheld survey data was available. Five more individuals were excluded as no online survey data was available. Comparing those participants who were included with those who were excluded from the data analyses revealed no relevant differences in terms of age, education level, BMI, past exercise behavior, or baseline scores on affective attitudes toward exercise (*p*s > 0.10). More women than men were excluded from the study (*p* < 0.05).

### Data Analyses

#### Preparatory Data Reduction

As described in Section “Procedures,” two measurements for the affective responses during an exercise session were applied. The first approximately between minutes 10 and 15 and the second between minutes 40 and 50. At both measurement points during an exercise session, participants answered questions in respect of two items per affective dimensions. The retest-reliabilities of the two measurements of all affective dimensions within the same exercise session were satisfactory to good (affective valence: 0.62 ≤*r* ≤ 0.75; calmness: 0.60 ≤*r* ≤ 0.69; positive activation: 0.57 ≤*r* ≤ 0.59). For further analyses, we merged the four answers on items per affective dimension during the same exercise session.

We also had two measurements per exercise session for the perceived exertion. These two data items were then combined as well to provide the mean exertion per exercise session. The two perceived exertion scores’ correlation during the exercises was satisfactorily strong (0.46 < *r* < 0.49).

#### Latent Change Scores

The change score for affective attitudes toward exercise (post-test–pre-test) was defined directly as latent variable (Geiser, 2010; Geiser et al., 2010).^2^ LC models can be used to compute LC variables that represent the inter-individual differences in intra-individual changes. The advantage of LC models is that the assessment of change in a latent construct can be controlled for measurement errors (Steyer et al., 1997).

The change variable was checked for strong factorial invariance. Factorial invariance is considered to be strong, when not only the (unstandardized) factor loadings of the manifest indicators remain constant across the different measurement times, but also the intercepts of the manifest indicators (Widamann and Reise, 1997). Furthermore, the model was checked for indicator-specific effects. Indicator specificity is seen as a common variance of the measurement error of an indicator across the different measurements (Eid et al., 1999). If indicator-specific effects are not taken into account, models might be misspecified and parameter estimates biased (Reuter et al., 2010).

The LC model was calculated using Mplus 5.21 (Muthén and Muthén, 1998–2010) and applying a maximum likelihood estimator. Missing values for the indicators of affective attitudes (59 missing values, 9.3%) were estimated by means of the full information maximum likelihood (FIML) procedure (Little and Rubin, 2012), as implemented in Mplus. The factor scores for affective attitudes at pre-test as well as for the LC score (post-test–pre-test) were appended to the analysis data set. Thereby, the factor scores can be considered as observed variables in the main analyses.

#### Two-Level Path Analysis

A two-level path analysis was carried out using Mplus 5.21 and applying a robust maximum likelihood estimator. With this analytic procedure, the hierarchical structure in the data can be taken into consideration. In this way it is possible to split up the total variance into one test person’s variance across the three sessions (within-person level) and the inter-individual variance (between-person level). On both model levels, all three affective dimensions were simultaneously taken into account. As the affect dimensions are known to be systematically interrelated (Schimmack and Grob, 2000; Wilhelm and Schoebi, 2007), correlations among these variables are included on both model levels at every given measurement point (pre-session, during session).

On the within-person level (level 1), we examined whether perceptions of competence and perceived exertion predict the intra-individual variance of affective responses across various exercise sessions. We controlled these effects for the pre-session values of the respective affective dimensions (autoregressive model), as well as for the situationally perceived fitness.

On the between-person level (level 2), we examined whether perceptions of competence and perceived exertion predict the inter-individual variation of affective responses during physical exercises. The focus was on general, cross-situational effects. The effects were then again controlled for the affective state prior to exercising, as well as for the perceived fitness. Based on already existing results (Sudeck and Conzelmann, 2014), we only tested if perceived fitness predict positive activation and affective valence during physical exercises. However, we did not examine whether perceived fitness predict calmness. All exogenous predictor variables were grand-mean centered.

We analyzed *the direct and indirect effects of exercise experiences on the changes in affective attitude* on the between-person level: firstly, it was tested whether perceptions of competence and the perceived exertion are directly related to changes in affective attitude. Secondly, we tested whether perceptions of competence and the perceived exertion are indirectly related to changes in affective attitude, i.e., imparted via the affective response during exercise. The critical significance level for path coefficients was set conventionally at α_crit_ = 0.05 (two-tailed). In order to test mediator effects on the between-person level, we performed bias-corrected bootstrap analyses (MacKinnon et al., 2004). Using this method, confidence intervals were estimated for indirect effects. If a 95% confidence interval does not contain zero, the indirect effect is significant at the 0.05 level.

Prior to carrying-out of the multi-level analysis, it was checked whether the level-specific proportions of the variance favor the application of a multi-level analysis. Intra-class coefficients (ICCs) were calculated for those variables which had been collected several times before, during, or after the exercise sessions. The ICC describes the relation of between-person variance to total variance. The ICC may take a value between 0 and 1. High values indicate a substantial between-person variance and provide an argument in favor of a two-level path analysis (Muthén, 1989).

**Figure 2** shows that due to absence some individuals were not able to participate in all handheld surveys in the exercise sessions. In total, the 105 participants of the reduced sample carried out 210 handheld surveys (80 in survey A, 68 in survey B, and 62 in survey C). These handheld surveys were filled out almost completely (see **Table 2**). Missing handheld survey data within the longitudinal data set were estimated again by means of the FIML procedure. In particular, data was imputed on those individuals not able to participate in all handheld surveys in the exercise sessions for reasons of absence. These missing observations were only replaced, however, where we had complete sets for both affective attitude data collections (pre-test and post-test) for the individual concerned. Under these conditions, 61 handheld computer surveys were replaced based on the model (22.5% of 271 observations; 16.1% of the data points of the two-level path model). By imputing the missing observations, and raising the number of observations up to 271, it was possible to strengthen the power of the longitudinal analysis.^3^

**Table 2:** Descriptives and intra-class coefficients of the measures of the handheld computer surveys in the exercise sessions (without missing value imputation, maximum: 210 observations).

	Number of observations	*M*	*SD*	Minimum	Maximum	Skewness	Kurtosis	ICC
Valence pre session	207	4.03	1.14	0	6.0	-0.41	0.02	0.351
Valence during session	210	4.62	0.86	2.0	6.0	-0.63	0.29	0.427
Pos. Activation pre session	207	3.48	1.12	0.5	6.0	-0.03	-0.66	0.308
Pos. Activation during	210	4.18	0.96	0.5	6.0	-0.71	1.31	0.406
Calmness pre session	207	3.56	1.28	0	6.0	-0.28	-0.25	0.531
Calmness during session	210	4.18	0.86	1.5	6.0	-0.13	-0.16	0.423
Perceived exertion	205	3.81	1.08	1.0	6.5	-0.14	-0.21	0.513
Perceptions of competence	210	3.96	0.88	1.0	5.0	-0.80	0.57	0.422
Perceived fitness	208	2.63	0.93	0	5.0	-0.16	0.09	0.701

The goodness of model fit was assessed through χ^2^ tests, comparative fit indices (CFI) and root-mean-square errors of approximation (RMSEA). A good model fit is characterized by a low χ^2^ value in relation to the degrees of freedom (χ^2^/*df* ≤ 2), as well as a high CFI ≥ 0.97 and a low RMSEA ≤ 0.05. A model fit is considered to be still acceptable when it has a χ^2^/*df*-ratio ≤ 3, as well as a CFI ≥ 0.95 and an RMSEA ≤ 0.08 (Schermelleh-Engel et al., 2003).

## Results

### Preliminary Analyses

#### Descriptive Results

**Table 2** shows the descriptive values of the exercise experiences (without missing value imputation; 210 observations). All three affective dimensions showed an increase in their mean values from the pre-session measurements to the measurements during the session. The skewness and kurtosis for the affective state measurements ranged from -1 to +1 respectively, with a slight exception for positive activation during exercising. Consequently, the majority of indicators did not diverge from the normal distribution.

The perceived exertion came in with a mean value of *M* = 3.80 (*SD* = 1.09; *Minimum* = 1; *Maximum* = 6.5), indicating a moderate degree of exertion. The clear majority of 85% of the ratings were ‘rather light’ to ‘somewhat hard’ (2.5 ≤ Perceived Exertion_during_ ≤ 5.5). The mean values for perceptions of competence were rather high (*M* = 3.96; *SD* = 0.88; *Minimum* = 1; *Maximum* = 5). Neither the perceived exertion nor perceptions of competence displayed any problematical deviations from the normal distribution (see **Table 2**). The same applied to the perceived fitness.

#### Preparatory Analyses for the Two-Level Path Analysis

The measuring properties of the LC score for affective attitudes toward exercise were good [χ^2^(10) = 12.85; *p* = 0.23; χ^2^/*df* = 1.29, CFI = 0.993; RMSEA = 0.052]. The estimated intercept of the pre-test score was *M* = 6.10 (*SE* = 0.19). The estimated mean value (*M* = 1.06, *SE* = 0.21) and variance (*Var* = 3.53, *SE* = 0.61) of the LC score deviated significantly from zero (*p* < 0.05). It is, therefore, justified to assume inter-individual differences in the LC score. Correlated measuring errors had to be considered for two indicators (AFF1: *r* = 0.52, *p* < 0.05; AFF3: *r* = 0.29, *p* < 0.05). Overall, these results justify the use of the factor scores for affective attitudes at pre-test, as well as for the LC scores as observed variables in the main analysis.

The ICCs of the used variables are listed in **Table 2**. The ICCs of the affective state values ranged from 0.30 to 0.47. The height of their values suggests that a multi-level analysis is appropriate. The ICC values for affective valence and positive activation during the session were higher than those prior to the session (12–15% higher variance between persons). The ICC values for calmness prior to and during the sessions were considerably closer to each other. Taken together, this intra-individual data indicate that the affective states during exercising were moderately consistent.

Perceptions of competence and the perceived exertion showed similar ICC values to the affective responses during the physical exercises. While for the perceived fitness, we could observe a higher intra-individual consistency.

### Two-Level Path Analysis: Indirect and Direct Link between Exercise Experiences and Changes in Affective Attitude toward Exercise

**FIGURE 3:**
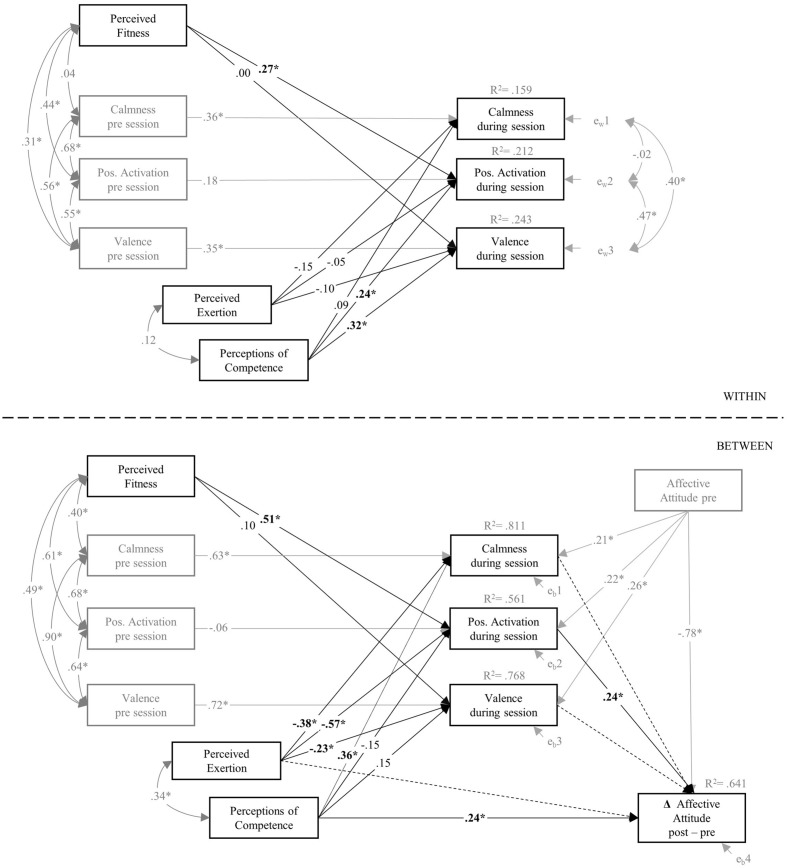
**Final results of the two-level path analysis.** All reported path coefficients (^∗^*p* < 0.05) are standardized estimates. Non-significant path coefficients for the prediction of Δ affective attitudes were not included in the final model (dotted lines; see main text for further information). For a better overview, correlations among the residuals of the affective states during the exercise session on the between-level of the model (*e_b_1, e_b_2, e_b_3*) were not illustrated. For the same reason, estimated intercepts, variances, and residual variances were not shown, which were estimated in line with the Mplus 5.21 default settings for the analysis TYPE = TWOLEVEL. At the within-person level, variances of the six exogenous variables as well as the residual variances of the three endogenous variables were estimated. At the between-person level, means and variances for the seven endogenous variables were estimated. Intercepts and residual variances were estimated for the four endogenous variables.

The global model fit of the initial two-level path analysis was acceptable to good [χ^2^(40) = 78.17; *p* = 0.0003; χ^2^/*df* = 1.95, CFI = 0.945; RMSEA = 0.059]. However, local model parameter estimates indicated suppression effects for the predictors of changes in affective attitude.^4^ Therefore, an alternative model specification appeared necessary in order to achieve a more parsimonious and interpretable model. As the interrelations of the three affect dimensions are theoretically reasonable, it was decided to eliminate critical components of the model as recommended by Maassen and Bakker (2001). For this purpose, non-significant path coefficients were stepwise eliminated from the initial model, which is a common procedure in exploratory path analyses (Pedhazur, 1982; Mertler and Vannatta, 2002). By doing so, three non-significant path coefficients have not been included in the final model (see dotted lines in **Figure 3**).

**Figure 3** shows the results of the two-level path analysis for the final model. The global fit of this reduced model was again acceptable to good [χ^2^(43) = 77.13; *p* = 0.0011; χ^2^/*df* = 1.79, CFI = 0.951; RMSEA = 0.054].

The relationship between perceived exertion, perceptions of competence and affective responses revealed different results patterns when comparing the two levels of the model: at the within-person level, only perceptions of competence was positively related to the affective response to exercising. In particular, perceptions of competence were associated with within-person variations of positive activation and affective valence across different exercise sessions. At the between-person level, both perceptions of competence and perceived exertion were related to a mean affective response to exercising. The perceived exertion was negatively related to all the affective dimensions. Perceptions of competence were positively associated to calmness during the exercise sessions. In addition, the perceived fitness level predicted within-person variations, as well as between-person variations of positive activation during the exercise sessions.

At the between-person level, direct effects on changes in affective attitude were found for positive activation during the exercise sessions (β = 0.24), as well as for perceptions of competence (β = 0.24). The final model on the between-person level revealed one indirect pathway between perceived exertion and changes in affective attitude via positive activation. This indirect path reached statistical significance (-0.14 [CI 95%: -0.26; -0.01]). The other indirect pathway between perceptions of competence and changes in affective attitude via positive activation did not reach statistical significance (-0.04 [CI 95%: -0.12; 0.05]). In total, the model could explain 64.1% of the variance of the LC scores of affective attitudes toward exercise.

## Discussion

In the present study, the relationship between exercise experiences and changes in affective attitude was investigated over a period of 13 weeks. The results provide important clues for a more detailed formulation of the transdisciplinary framework model on changing exercise behavior put forward by Bryan et al. (2011).

Our results partially support the assumption of an indirect effect made by Williams (2008), who assumed that cognitive and interoceptive factors predict affective responses during exercising, and that these in turn predict affective attitudes (see **Figure 1**). The results in fact confirm the existence of a chain of effects stretching from perceived exertion via positive activation during exercising to changes in affective attitude. On average, the less strenuous people perceive physical exercise to be, the more awake they will feel during exercise. Those who on average reported more positive activation also showed more improvement in affective attitudes from the beginning to the end of the exercise program. Contrary to this, no indirect relationship between perceptions of competence and changes in affective attitude could be found. In fact, the average level of perceived competence is directly linked to changes in affective attitude without having to be fully reproduced in the affective response to exercise. This direct effect of perceptions of competence illustrates the importance of cognitive factors with respect to changes in affective attitudes toward exercise. Our study thus provides empirical evidence on how affective attitudes to exercising changes depending on interoceptive, affective, and cognitive factors. *In situ* exercise experiences especially may be identified as an important source of changes in affective attitude.

While exercise experiences in existing studies were recorded retrospectively, the data for this study were obtained *in situ* and across several sessions. These procedures made it possible to specify the relationship between individual facets of exercise experiences. Furthermore, they allowed us to substantiate the relationship between exercise experiences and affective attitudes:

(1) It became evident that inter-individual differences in the perceived exertion has a strong negative relationship with affective responses to physical exercise. This is particularly true for the psycho-physiologically determined affective dimension of positive activation as well as for calmness. This result is especially interesting as the intensity of physical strain predominantly ranged from moderate to somewhat hard. According to the dual mode theory, however, inter-individual differences in affective responses should primarily appear with higher intensities (i.e., with strenuous physical exercises, Ekkekakis and Acevedo, 2006).
(2) Moreover, the negative indirect relationship between average levels of perceived exertion on affective attitudes via positive activation during exercise is in line with previous findings on the role of negative physical experiences on affective behavioral predictors. In their diary study, Loehr et al. (2014) identified that body soreness and the perception of exercise as being a chore were negatively associated with positive affective outcome expectancies. The indirect effect in our study indicates that those negative physical experiences which are linked to interoceptive factors during exercise are associated with lower positive activation while exercising. Therefore, the connection between negative physical experiences and affective behavioral predictors may be partly mediated by affective responses to physical exercise.
(3) By means of the two-level analysis, it became evident that the connections between individual exercise experiences are level-specific. While perceived exertion was not connected with intra-individual variations in affective responses, negative relations to the inter-individual variance in affective responses resulted. One possible explanation for inter-individual differences in the affective responses to exercise could be seen in the varying preferences and tolerances for exercise intensities (Ekkekakis et al., 2005). According to this concept, dispositional inter-individual differences exist with respect to self-chosen intensities (preferences) and as to what extent higher strains could be tolerated (tolerance). Such dispositional differences would provide support for a connection between perceived exertion and inter-individual variation in affective responses in the examined range of intensity. Relationships between physical exertion and intra-individual variations, on the other hand, would be expected to a lesser extent.
(4) This study confirms that perceptions of competence are particularly significant for affective responses to exercising (e.g., Rose and Parfitt, 2012). Perceptions of competence not only predicted intra-individual but also inter-individual differences in affective responses when exercising. However, to which affective dimension the responses were attached depends on the level of analysis. Intra-individual variations in both positive activation and valence seem to be positively affected by situational competence experiences; in contrast to that, inter-individual differences in calmness during physical exercises seem more strongly positively associated with the average level of competence experiences. This explorative analysis of specific links between competence experiences and affective dimension would have to be tested and theoretically elaborated in future studies.

Overall, our study supports the assumption that a closer view of exercise experiences contributes to theory development on changes of exercise behavior with a special focus on affective predictors of behavior. For example, the relationships found between average levels of exercise experiences and changes in affective attitude toward physical exercise may, as explained above, be the result of a direct effect. In this way, the robust direct and positive effect of the average level of perceptions of competence on affective attitudes toward exercise demonstrates the importance of the cognitive appraisal of exercise experiences for future affective attitudes toward exercise. However, alternative cognitive mechanisms are also conceivable, such as those regarding the sources of self-efficacy (Samson and Solmon, 2011). It is possible that perceptions of competence, as a kind of mastery experience, have an impact on affective attitudes via self-efficacy (Gellert et al., 2012). Further, it may be assumed that perceptions of competence, as well as low perceived exertion, lead to positive implicit associations with physical exercise. These subconscious associations could in turn be the underlying reason for explicit, affective attitudes (Bluemke et al., 2010). To review alternative effect mechanisms, it is necessary to include further motivational constructs in future examinations [e.g., self-efficacy (Kwan and Bryan, 2010; Schneider and Kwan, 2013); affective outcome expectancies and satisfaction (Baldwin et al., 2013; Loehr et al., 2014)]. In doing so, one could further elaborate on the potential of affective predictors of the adoption and maintenance of physical exercises in comparison to other motivational and volitional predictors of behavior, which were already established in other studies [e.g., outcome expectancies, self-efficacy, intention (Gellert et al., 2012); intention (Mohiyeddini et al., 2009); outcome expectancies, fulfillment of outcome expectancies, action self-efficacy, (Klusmann et al., 2016)].

### Limitations

The present study includes some methodical limitations which must be recognized:

(1) In the main analysis, a problem with suppression effects appeared in connection with the prediction of changes in affective attitude. This was probably due to the fact that the model covered three affect dimensions that are systematically interrelated (Schimmack and Grob, 2000). The model’s specification had to be slightly modified so that the final model test was no longer, in the strict sense, confirmatory. The results of the final model revealed that average levels of positive activation had the strongest relationship to changes in affective attitude when compared to the other affect dimensions. This pattern of results is in line with the bivariate correlation matrix (see Supplementary material). However, the particular role of positive activation during exercise for the prediction of medium-term changes in affective attitude needs further reinforcement by purely confirmatory analyses in the future.
(2) In our study, exercise experiences were repeatedly obtained *in situ* in natural surroundings, and these were linked to medium-term changes of affective attitude toward exercise. The study thus has a high ecological validity. Nonetheless, it should be noted that the scope of application of the results is limited to *combined* exercise programs in the recreational and health sector. Such exercise programs, in which endurance, strength training, and playful activities are combined in a single session, are very widespread in German-speaking countries (Brehm et al., 2005).
(3) We could not examine whether the study’s results varied for the different exercise programs. The sub-samples were too small for a differentiated analysis of the programs’ contents and exercise types. For this reason, the findings may, for the time being, only be generalizable to exercises which require physical exertion ranging from moderate to rather strenuous and are carried out in a group and under guidance.
(4) The examined sample included some specific characteristics. We recruited university employees for whom we developed the specific programs. This again limits the generalizability of the findings. Furthermore, the sample was already comparatively active: 50% of the subjects did at least 2 h of physical exercise a week. It would be interesting if future studies could focus more on inactive people or people with increased health risks. Additionally, in the present study more men than women could be considered in the longitudinal analysis. Whether gender represents a moderator for the effect mechanisms remains to be tested.
(5) In our study, it was shown that exercise experiences were moderately consistent at the intra-individual level. Obviously, this degree of consistency was sufficient to make predictions about changes in affective attitude over a 3-months period. What is striking is the fact that the observed intra-individual consistencies in exercise experiences were lower than those reported by Unick et al. (2015). Two reasons for this can be provided: firstly, the physical exercises in the present study were distinctly less standardized. Indeed, the course instructors were instructed to conduct typical sessions. Nevertheless, distinctly larger differences, as far as content and social conditions are concerned were possible between these sessions than in the case of treadmill exercises. Secondly, in the present study, handheld computer surveys were conducted during different weeks in the course of the exercise program. Differences between participation in the first sessions and later sessions of the exercise program may have had an effect on intra-individual differences in the experience of (the same) physical exercises.
(6) Finally, there are some drawbacks in the way the constructs of interest were measured. The questionnaires used to measure perceptions of competence and physical exertion only contained a single item. However, the connections found between the variables suggest that the construct validity of the procedures was satisfactory.

## Conclusion

Which research desiderata can be deduced from our study? In future studies, the point in time over the course of an exercise program should be systematically considered and examined. Particularly for new participants in exercise programs, an analysis of temporal patterns would be valuable for understanding their experience of physical exercise over the course of the sessions, as such temporal patterns might exhibit a connection to their future exercise behavior. A point in favor of this assumption is the positive relationship between physical fitness and positive activation during exercising (cf. Ekkekakis et al., 2011). Obviously, previous exercise experiences and the connected physical fitness are associated with affective and motivational processes. Thus, future studies should apply systematic recruitment strategies that consider previous exercise behavior in order to be able to analyze the moderating role of previous exercise behavior with sufficient sample sizes of subgroups.

Furthermore, it would be efficacious to use more intensive data acquisition strategies that better reflect the in-depth dynamics of the interplay between different exercise experiences, affective predictors of behavior, and exercise behavior.

Finally, the present study only examined one link of the postulated chain of effects, in the form of exercise experiences and affective attitudes (see **Figure 1**). Future studies should expand this perspective. It would be particularly interesting to take behavior into account as well. Thus, the question might be asked here too, whether affective responses influence exercise behavior indirectly, via motivation – as suggested by Bryan et al.’s (2011) transdisciplinary framework model or whether they have a direct impact on behavior (Williams et al., 2012).

Overall, this study makes clear that perceptions of competence, perceived exertion and affective responses during physical exercise play an important role in affective processes of behavior change. It appears that the average levels of these experiences predict changes in affective attitude during multi-week exercise programs. However, it is recommended that future studies with a higher sample size replicate the main findings obtained for these between-person relationships. For now, our study supports the idea that interventions which aim to change exercise behavior should put more emphasis on exercise experiences in order to address affective behavioral predictors.

## Author Contributions

GS and AC contributed to the study design. Data collection and data analysis was performed by GS and JS. All authors contributed to the interpretation of the data analysis. GS and JS drafted the manuscript, and AC provided critical revisions. All authors approved the final version of the manuscript for submission. All authors agree to be accountable for all aspects of the work ensuring that questions related to the accuracy or integrity of any part of the work are appropriately investigated and resolved.

## Conflict of Interest Statement

The authors declare that the research was conducted in the absence of any commercial or financial relationships that could be construed as a potential conflict of interest.

## Footnotes

1 It is important to note that the different types of exercises in the exercise programs are not a consideration in this work. With regard to the affective responses, we focused on physical exercises taking place in a natural, non-laboratory testing environment. Affective responses to techniques of relaxation are not taken into consideration. As such techniques of relaxation were systematically carried out at the end of the sessions in two out of the five programs, the affective states at the end of the sessions were not included in the analyses.

2 A full presentation of the LC model can be found in the Supplementary material.

3 For comparison, we calculated a two-level path analysis exclusively using those cases for which the handheld computer surveys from the exercise sessions were obtained (210 observations). The results were not substantially different.

4 The following typical pattern of results indicating a suppression effect (Maassen and Bakker, 2001) was observed: firstly, a high negative standardized path coefficient occurred for calmness during the session (β = -0.62; *SE* = 1.08, *p* = 0.57). Secondly, positive activation during the session had a rather high positive path coefficient (β = 0.40; *SE* = 0.33, *p* = 0.25) that was higher than the bivariate correlation between positive activation during the session and the change score for affective attitudes (see between-person correlation matrix in the Supplementary material). Thirdly, the residual correlations among the three affective dimensions during the exercise session were very high (0.83 < *r* < 0.96), which correlates with high values in the between-person correlation matrix. Additionally, all standard errors were rather high when compared to the other parts of the two-level path analysis. In detail, the further standardized path coefficients for the prediction of change scores in affective attitudes were as follows: valence during session: β = 0.27; *SE* = 0.67, *p* = 0.68; perceived exertion: β = -0.11; *SE* = 0.24, *p* = 0.65; perceptions of competence: β = 0.43; *SE* = 0.37, *p* = 0.25.
