# Supplementary material for: Exercise Experiences and Changes in Affective Attitude: Direct and Indirect Effects of In Situ Measurements of Experiences
Source: Front Psychol. 2016 Jun 16;7:900. doi: 10.3389/fpsyg.2016.00900 (PMC4909746; doi:10.3389/fpsyg.2016.00900)
Supplement: Supplementary file 1 [file Table_1.DOCX]

Supplementary Material

**Exercise experiences and changes in affective attitude:
Direct and indirect effects of *in situ* measurements of experiences**

**Gorden Sudeck^1^*, Julia Schmid^2^ and Achim Conzelmann^2^**

^1^Institute of Sport Science, University of Tübingen, Tübingen, Germany

^2^Institute of Sport Science, University of Bern, Bern, Switzerland

*** Correspondence:** Gorden Sudeck, Institute of Sport Science, University of Tübingen, Wilhelmstraße 124, 72074 Tübingen, Germany**;** [gorden.sudeck@uni-tuebingen.de](mailto:gorden.sudeck@uni-tuebingen.de)

# Supplementary Figures and Tables

## Supplementary Figures


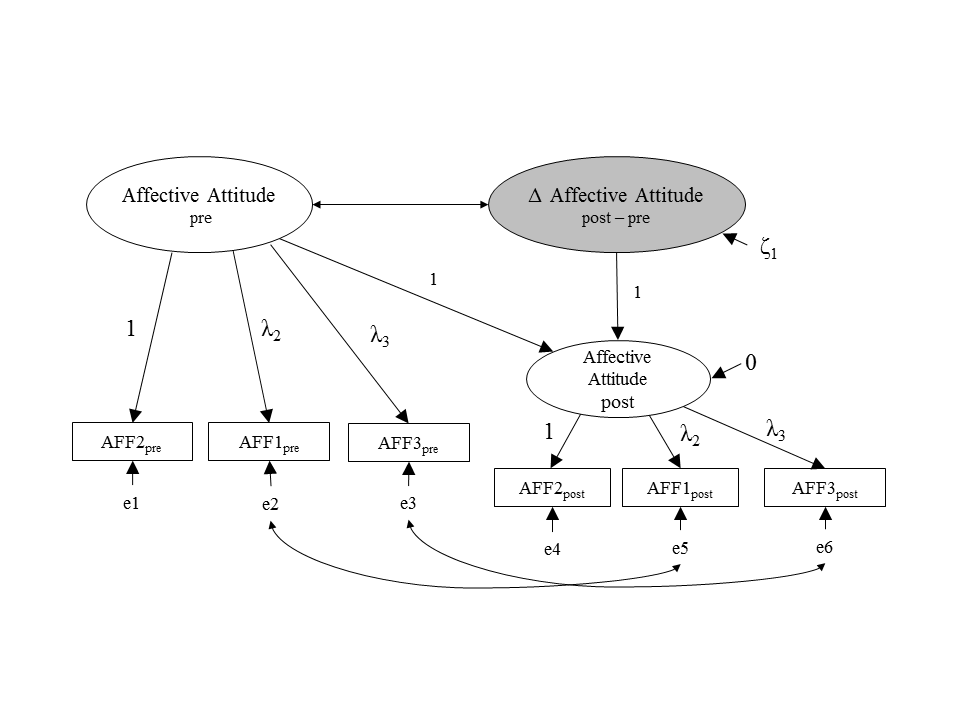


**Supplementary Figure 1.** Model specification for the latent change model for affective attitudes toward exercise

**Note.** The latent change modelling was conducted in accordance to Geiser (2010). Intercepts for the manifest indicators *AFF1_pre_, AFF3_pre_, AFF1_post_*, and *AFF3_post_* are not shown but were estimated. In contrast, intercepts of the manifest indicators *AFF2_pre_* and *AFF2_post_* are fixed to zero in order to enable the estimation of the means of the according latent factors. Moreover, factor variances for affective attitude at pre test and for Δ affective attitude were estimated. Additionally, residuals variances for *e1* to *e6* were estimated by default in Mplus 5.21.

## Supplementary Table

## Supplementary Table 1. Within-person correlation matrix for the two-level path analysis

|  | VA_PRE_ | VA_DUR_ | PA_PRE_ | PA_DUR_ | CA_PRE_ | CA_DUR_ | PC | RPE | FIT |
| --- | --- | --- | --- | --- | --- | --- | --- | --- | --- |
| VA_PRE_ | 1.00 |  |  |  |  |  |  |  |  |
| VA_DUR_ | 0.42 | 1.00 |  |  |  |  |  |  |  |
| PA_PRE_ | 0.53 | 0.33 | 1.00 |  |  |  |  |  |  |
| PA_DUR_ | 0.29 | 0.56 | 0.41 | 1.00 |  |  |  |  |  |
| CA_PRE_ | 0.54 | 0.21 | 0.20 | 0.16 | 1.00 |  |  |  |  |
| CA_DUR_ | 0.19 | 0.42 | 0.07 | 0.08 | 0.35 | 1.00 |  |  |  |
| PC | 0.26 | 0.42 | 0.24 | 0.30 | 0.09 | 0.14 | 1.00 |  |  |
| RPE | -0.04 | -0.15 | -0.06 | -0.16 | 0.10 | -0.11 | -0.16 | 1.00 |  |
| FIT | 0.30 | 0.13 | 0.43 | 0.37 | 0.04 | 0.03 | 0.06 | -0.15 | 1.00 |

**Abbreviations.** VA_PRE_ = Valence pre session; VA_DUR_ = Valence during session; PA_PRE_ = Positive Activation pre session; PA_DUR_ = Positive Activation during session; CA_PRE_ = Calmness pre session; CA_DUR_ = Calmness during session, PC = Perceptions of Competence; RPE = Perceived Exertion; FIT = Perceived Fitness

**Supplementary Table 2.** Between-person correlation matrix for the two-level path analysis

|  | AFF_PRE_ | ΔAFF | VA_PRE_ | VA_DUR_ | PA_PRE_ | PA_DUR_ | CA_PRE_ | CA_DUR_ | PC | RPE | FIT |
| --- | --- | --- | --- | --- | --- | --- | --- | --- | --- | --- | --- |
| AFF_PRE_ | 1.00 |  |  |  |  |  |  |  |  |  |  |
| ΔAFF | -0.71 | 1.00 |  |  |  |  |  |  |  |  |  |
| VA_PRE_ | -0.20 | 0.19 | 1.00 |  |  |  |  |  |  |  |  |
| VA_DUR_ | 0.19 | 0.04 | 0.63 | 1.00 |  |  |  |  |  |  |  |
| PA_PRE_ | -0.06 | 0.19 | 0.68 | 0.20 | 1.00 |  |  |  |  |  |  |
| PA_DUR_ | 0.17 | 0.18 | 0.13 | 0.67 | 0.00 | 1.00 |  |  |  |  |  |
| CA_PRE_ | -0.13 | 0.04 | 0.91 | 0.59 | 0.66 | 0.05 | 1.00 |  |  |  |  |
| CA_DUR_ | 0.15 | 0.05 | 0.40 | 0.80 | 0.04 | 0.53 | 0.56 | 1.00 |  |  |  |
| PC | 0.19 | 0.16 | -0.31 | 0.16 | -0.32 | 0.14 | -0.32 | 0.35 | 1.00 |  |  |
| RPE | 0.10 | -0.30 | -0.22 | -0.44 | 0.14 | -0.48 | -0.25 | -0.64 | -0.27 | 1.00 |  |
| FIT | -0.01 | 0.22 | 0.49 | 0.52 | 0.61 | 0.50 | 0.40 | 0.33 | 0.20 | -0.08 | 1.00 |

**Note.** AFF_PRE_ = Affective Attitude pre; ΔAFF = Δ Affective attitude post – pre; VA_PRE_ = Valence pre session; VA_DUR_ = Valence during session; PA_PRE_ = Positive Activation pre session; PA_DUR_ = Positive Activation during session; CA_PRE_ = Calmness pre session; CA_DUR_ = Calmness during session, PC = Perceptions of Competence; RPE = Perceived Exertion; FIT = Perceived Fitness
